# Fauna and seasonality of sand flies (Diptera: Psychodidae: Phlebotominae) from a leishmaniasis transmission area in the central region of Rio Grande do Sul, Brazil

**DOI:** 10.1590/S1984-29612024042

**Published:** 2024-08-12

**Authors:** Vanessa Osmari, Fagner D’ambroso Fernandes, Maurício Tatto, Getúlio Dornelles Souza, Fabiana Raquel Ratzlaff, Jaíne Soares de Paula Vasconcellos, Sônia de Avila Botton, Diego Willian Nascimento Machado, Fernanda Silveira Flores Vogel, Luís Antônio Sangioni

**Affiliations:** 1 Laboratório de Doenças Parasitárias – LADOPAR, Departamento de Medicina Veterinária Preventiva – DMVP, Centro de Ciências Rurais – CCR, Universidade Federal de Santa Maria – UFSM, Santa Maria, RS, Brasil; 2 Centro Universitário Ritter dos Reis – UniRitter, Porto Alegre, RS, Brasil; 3 Laboratório de Reservatórios e Vetores, Laboratório Central do Estado do Rio Grande do Sul, Porto Alegre, RS, Brasil; 4 Laboratório de Saúde Única – LASUS, Departamento de Medicina Veterinária Preventiva – DMVP, Centro de Ciências Rurais – CCR, Universidade Federal de Santa Maria – UFSM, Santa Maria, RS, Brasil; 5 Programa de Pós-graduação em Geociências, Instituto de Geociências, Universidade Federal do Rio Grande do Sul – UFRGS, Porto Alegre, RS, Brasil

**Keywords:** Entomology, sand flies, leishmaniasis, One Health, epidemiology, Santa Maria, Entomologia, flebotomíneos, leishmaniose, Saúde Única, epidemiologia, Santa Maria

## Abstract

Sand flies, vectors capable of transmitting *Leishmania* spp. and causing leishmaniasis, have been a concern in the central region of Rio Grande do Sul, where canine leishmaniasis (CanL) has been documented since 1985. Notably, there has been a surge in CanL cases since 2017, with two autochthonous cases of human visceral leishmaniasis reported in the area in 2021. This study aimed to identify the sand fly fauna potentially involved in disease transmission. Modified Centers for Disease Control light traps were deployed in three neighborhoods of the city where CanL cases had been previously reported, spanning January 2021 to December 2022. Of the 89 collections conducted, 119 sand flies belonging to five species were captured: *Pintomyia fischeri* (76/119, 63.86%), *Migonemyia migonei* (23/119, 19.33%), *Lutzomyia longipalpis* (16/119, 13.45%), *Brumptomyia* sp. (2/119, 1.68%), and *Psathyromyia lanei* (2/119, 1.68%), predominantly between February and April in 2021 and 2022. Polymerase chain reaction testing on all female specimens yielded negative results for *Leishmania* spp. DNA. Although Leishmania spp. was not detected in these vectors, these findings underscore the imperative to implement measures aimed at curtailing the proliferation of these insects.

## Resumo

Os flebotomíneos, vetores capazes de transmitir *Leishmania* spp. e causar leishmaniose, têm sido uma preocupação na região central do Rio Grande do Sul, onde a leishmaniose canina (CanL) é documentada desde 1985. Notavelmente, houve um aumento nos casos de CanL desde 2017, com dois casos autóctones de leishmaniose visceral humana relatados na área, em 2021. Este estudo teve como objetivo identificar a fauna de flebotomíneos potencialmente envolvida na transmissão da doença. Armadilhas luminosas modificadas, do Centro de Controle de Doenças, foram implantadas em três bairros da cidade onde casos de CanL haviam sido relatados anteriormente, no período de janeiro de 2021 a dezembro de 2022. Das 89 coletas realizadas, 119 flebotomíneos de cinco espécies foram capturados: *Pintomyia fischeri* (76/119, 63,86%), *Migonemyia migonei* (23/119, 19,33%), *Lutzomyia longipalpis* (16/119, 13,45%), *Brumptomyia* sp. (2/119, 1,68%) e *Psathyromyia lanei* (2/119, 1,68%), predominantemente, entre fevereiro e abril de 2021 e 2022. Testes de reação em cadeia da polimerase em todos os espécimes fêmeas resultaram negativos para DNA de *Leishmania* spp. Embora *Leishmania* spp. não tenha sido detectada nesses vetores, esses achados destacam a necessidade imperativa de implementar medidas destinadas a conter a proliferação desses insetos.

## Introduction

There are 1,026 species of Phlebotominae known worldwide, 539 in the Americas and 277 in Brazil (Galati, 2018). In Rio Grande do Sul (RS), 23 species have been identified (Silva et al., 2004; Andrade et al., 2007). Sand flies are insects in the order Diptera, subfamily Nematocera, family Psychodidae, and subfamily Phlebotominae. Some species in this subfamily are vectors for *Leishmania* spp. causing human visceral leishmaniasis (HVL), canine leishmaniasis (CanL), and Cutaneous Leishmaniasis (CL) (Dantas-Torres, 2009).

Brazil has the highest number of cases in the American continent (WHO, 2017). Visceral leishmaniasis (VL) in Brazil is primarily caused by *Leishmania infantum*, transmitted by *Lutzomyia longipalpis* and *Lutzomyia cruzi* (Queiroz et al., 2012). Other species implicated in transmission include *Nyssomyia neivai*, *Pintomyia fischeri,* and *Migonemyia migonei* (Guimarães et al., 2016).

In urban areas, dogs (*Canis familiaris*) are the main infection reservoirs and sources. In the wild, foxes (*Dusicyon vetulus* and *Cerdocyon thous*) and marsupials (*Didelphis albiventris* and *Didelphis marsupialis*) also serve as reservoirs (Brasil, 2014). Studies have shown bat involvement in the protozoan lifecycle (Ratzlaff et al., 2022). In the state of RS, the first autochthonous cases occurred in dogs in 2008 and in humans the following year, with *L. longipalpis* identified as the main vector (Brasil, 2014).

According to the State Department of Health of Rio Grande do Sul, eight municipalities registered the presence of *L. longipalpis* until 2017. However, the municipalities of Viamão, Porto Alegre, and Santa Cruz do Sul did not register the main vector, with cases occurring in dogs and humans. The affected individuals lived close to forest fragments, and transmission was attributed to sand flies belonging to wild fauna (Rio Grande do Sul, 2017). In Porto Alegre, 777 CanL cases were reported between 2010 and 2021, and 20 HVL cases were confirmed between 2016 and 2021 (Rio Grande do Sul, 2022).

Sporadic CanL cases have been reported in Santa Maria since 1985 (Pocai et al., 1998). However, as of 2017, there has been a considerable increase in CanL incidence (116 cases) and in 2021, two autochthonous human cases were recorded, one of which contributed to the patient’s death (Wille, 2021).

According to the VL and CL Control Programs, recommended by the Ministry of Health, the objective of entomological investigations is to collect quantitative and qualitative information on transmitting sand flies in order to obtain new knowledge of the bioecology of insect species pertinent to human health (Brasil, 2014). Therefore, this study aimed to identify sand fly fauna potentially involved in the transmission of leishmaniasis, in a disease transmission area in the interior of the state of RS, and to present data related to the seasonal behavior of the main species found.

## Materials and Methods

**Figure 1:**
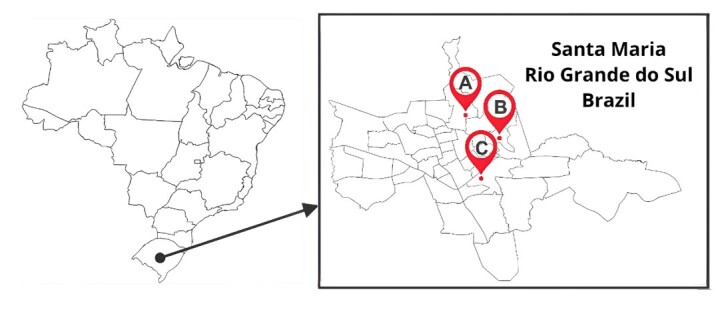
Study location. (A) Nossa Senhora do Perpétuo Socorro; (B) Presidente João Goulart; (C) Cerrito.

The municipality of Santa Maria (Figure 1) is located in central Rio Grande do Sul, southern Brazil, at 29°41’02”S and 53°48’25”W and an altitude of 113 m. The city has an area of 1,780.194 km^2^ and a population of 285,159 inhabitants (IBGE, 2021).

To capture the insects, modified CDC traps (Horst™ model) were installed in locations with previous CanL notifications from the Environmental Surveillance of the Municipality of Santa Maria, as shown in Figure 1. These locations were visited to request authorization from residents to install traps. Thus, three different locations were selected: Nossa Senhora do Perpétuo Socorro in the North (A), Presidente João Goulart in the Northeast (B), and Cerrito in the Center-East region (C) (Table 1). All sites were selected based on confirmation of dogs that presented anti-*Leishmania* spp. antibodies in rapid tests (TR-DPP®) and indirect immunofluorescence assay (IFA) (Varandas et al., 2001), as well as traps were installed according to the availability of residents to monitor and on days with no precipitation or strong winds.

**Table 1:** Description of sampling points and species of sand flies captured in the neighborhoods Nossa Senhora do Perpétuo Socorro, Presidente João Goulart and Cerrito in Santa Maria, RS, Brazil.

Sampling location	Geographic coordinates	Environment description	Neighborhood/Region	Captured species
A	29°40’29.03’S	Predominantly urbanized environment, peridomicile with the presence of a stream surrounded by residual vegetation, abundant organic matter, humidity and presence of domestic cats	Nossa Senhora do Perpétuo Socorro (North Region)	*Lutzomyia longipalpis*
	53°48’38.04’W			
B	29°40’47.92”S	Predominantly urbanized environment, peridomicile with area shaded by bamboo trees (*Bambusa taquara*), organic matter and domestic dogs.	Presidente João Goulart (Northeast Region)	*Lutzomyia longipalpis Migonemyia migonei*
	53°47’29.07”W			
C	29°41’58.59”S	Peridomicile, adjacent to a vast area of well-preserved residual Atlantic Forest, rich in organic matter and the presence of both domestic animals and pheasants, as well as occasionally wild mammals (*Didelphis albiventris*).	Cerrito (Central-East Region)	*Pintomyia fischeri*
				*Migonemyia migonei*
				*Lutzomyia longipalpis*
				*Brumptomyia* sp.
	53°47’60.87”W			
				*Psathyromyia lanei*

The traps were installed in the peridomicile, which were evaluated: the presence of domestic animals, organic matter, undergrowth, and fruit trees with shaded areas. The collections were carried out in the period between January 2021 and December 2022. The frequency of installing the traps was at least once a month.

The traps were placed approximately 1.5 m above the ground, activated at dusk (06:00 pm), and removed at dawn the next day (06:00 am), remaining uninterrupted for 12 h, coinciding with the feeding habits of sand flies. The devices were allocated at least once per month to each selected household. The temperature, precipitation, and humidity for the study period were obtained from the Instituto Nacional de Meteorologia (INMET, 2022).

After removing the traps, the collection bags were sent to the Parasitic Diseases Laboratory (LADOPAR) of the Universidade Federal de Santa Maria (UFSM) for captured insect selection. To dispatch the captured arthropods, the collection bag was stored in a freezer at -20 °C for 30 min. After this, all insects were examined under a stereoscopic microscope (Olympus®), and sand flies were screened according to external morphological characteristics, including size, presence of bristles throughout the body, and lanceolate wings (Galati, 2018). The remaining insects were discarded.

The selected specimens were sent to the Reservoir and Vector Laboratory of the Central Laboratory of the State of Rio Grande do Sul for morphological identification according to the taxonomic key proposed by Galati (2018). The results were tabulated in Excel spreadsheets, where sex, species, subspecies, locality of origin, climatic conditions, and environmental characteristics were recorded.

For molecular analysis, female flies were separated according to the date, place, and species found in each collection bag, then divided into individual samples or pools of up to 10 specimens. The samples were extracted using the Purelink® Genomic DNA Mini Kit (Invitrogen, USA) following the manufacturer's recommendations. The assessment of DNA quality and quantity extracted was conducted using the NanoDrop 1000 Spectrophotometer and measuring absorbance at 260/280 nm (Thermo Fisher Scientific). Subsequently, all samples were stored at -20 °C v PCR was performed.

The primers leishmini-F:5′-GGKAGGGGCGTTCTGC-3′ and leishmini-R:5′-STATWTTACACCAACCCC-3′ were used to amplify 120 pb kinetoplast DNA (kDNA) minicircles (Kocher et al., 2018). The PCR reaction was prepared in a final volume of 10 µL containing 1 × buffer (Invitrogen, Carlsbad, CA, USA), 2 mM MgCl_2_, 0.2 mM dNTPs (Ludwig Biotec, Brazil), 0.2 µM of each primer (Exxtend Biotecnologia, São Paulo, Brazil), 2 U Taq DNA Polymerase (Invitrogen, Carlsbad, CA, USA), Milli-Q water, and approximately 20 ng of extracted DNA sample. Positive and negative controls were included in all reactions, consisting of DNA extracted from *L. infantum* culture (MHOM/BR/1974/PP75) and Milli-Q water, respectively.

Amplification was performed in an automatic thermal cycler (T100, Bio-Rad, Singapore) following the recommendations of Kocher et al. (2018) with the following conditions: initial denaturation at 95 °C for 10 min, denaturation at 95 °C for 30 s, followed by 35 cycles of annealing at 52 °C for 30 s, extension at 72 °C for 20 s, final extension at 72 °C for 5 min, and a final PCR cycle at 4 °C.

The amplification reaction products were subjected to electrophoresis in 3% agarose gel (Ludwig Biotecnologia, Brazil), stained with SYBR safe DNA (Invitrogen, USA), stained with a UV transilluminator, and photographed for analysis.

To confirm inhibition of the reaction in PCR-negative samples, 0.25 μL DNA from a positive control sample was added to 0.25 μL DNA from a negative sample, and PCR was performed under the same conditions mentioned above. Additionally, the positive control were also diluted in the serial dilutions from 1:10 to 1:10^4^ and retested, in order to determine the detection threshold.

## Results

From January 2021 to December 2022, 89 collections were conducted: 32 in the Nossa Senhora do Perpétuo Socorro neighborhood (A), 28 in Presidente João Goulart (B), and 29 in Cerrito (C). The total population of sand flies captured in the municipality of Santa Maria, RS during the study period is shown in Table 2.

**Table 2:** Species of sand flies collected with light traps of the CDC type from January 2021 to December 2022, in the neighborhoods Nossa Senhora do Perpétuo Socorro (A), Presidente João Goulart (B) and Cerrito (C), Santa Maria, RS, Brazil.

**Species**	A		B		C		Total (%)
**♂**	**♀**	**♂**	**♀**	**♂**	**♀**
*Pintomyia fischeri*	0	0	0	0	18	58	76 (63.86)
*Migonemyia migonei*	0	0	1	0	11	11	23 (19.33)
*Lutzomyia longipalpis*	3	2	2	4	1	4	16 (13.45)
*Brumptomyia* sp.	0	0	0	0	2	0	2 (1.68)
*Psathyromyia lanei*	0	0	0	0	0	2	2 (1.68)

A total of 119 sand flies of five species were collected: *P. fischeri* (76/119, 63.86%), *M. migonei* (23/119, 19.33%), *L. longipalpis* (16/119, 13.45%), *Brumptomyia* sp. (2/119, 1.68%), and *Psathyromyia lanei* (2/119, 1.68%). Of these, 38 (31.94%) were male and 81 (68.06%) were female. Specimens of the genus *Brumptomyia* could not be identified at the species level because of damage to morphological structures necessary for identification.

In the Nossa Senhora do Perpétuo Socorro neighborhood (A), there was a stream with vegetation on the banks, abundant organic matter, humidity, and domestic cats; only *L. longipalpis* was found. The Presidente João Goulart neighborhood (B) collection site had an area shaded by bamboo (*Bambusa taquara*), organic matter, and domestic dogs, one of which was seropositive for *Leishmania* spp. during the study. Although this disease was not the primary cause of death in dogs, *L. longipalpis* and *M. migonei* were found. However, the Cerrito neighborhood (C) is adjacent to a vast, well-preserved residual Atlantic Forest, rich in organic matter and containing both domestic and wild animals, including wild opossums (*Didelphis albiventris*) and pheasants (*Phasianus colchicus*). In this locality, five species of sand flies were identified.

**Figure 2:**
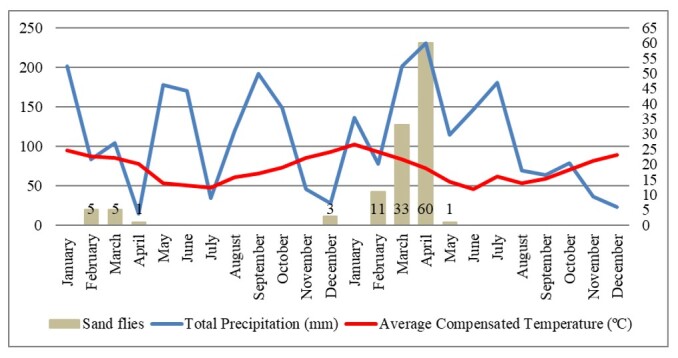
Seasonal distribution of sand flies (un), in relation to Average Compensated Temperature (°C) and Total Precipitation (mm), in the period between January 2021 and December 2022, in the municipality of Santa Maria, RS, Brazil.

A graphic representation of the seasonal distribution of sand flies collected in Santa Maria, RS, in relation to the average compensated temperature (°C) and total monthly precipitation between January 2021 and December 2022 is shown in Figure 2.

The largest number (108/119, 90.75%) of specimens was collected in February, March, April, and December 2021, as well as February, March, April, and May 2022. These periods preceded or coincided with high rainfall and average temperatures > 20 °C. However, in periods with little rain and consequently low relative humidity or average temperatures < 20°C, the months with the lowest amount or no sand flies collected occurred.

Of the 81 female flies captured and divided into 30 individual samples or pools, all were tested using PCR and were negative for *Leishmania* spp. DNA.

## Discussion

In the last 20 years, the southern region of Brazil has registered an increased number of CanL, and HVL cases (Rio Grande do Sul, 2022). Based on identification of the main vector (*L. longipalpis*), Souza et al. (2008) observed that protozoa were transmitted autochthonously in western Rio Grande do Sul. Furthermore, Rêgo et al. (2020) showed that in Porto Alegre, *P. fischeri*, *M. migonei,* and *L. gaminarai* may be associated with disease transmission in areas without *L. longipalpis*.

The main species transmitting VL in Brazil, *L. longipalpis*, was the third most abundant in this study (16/119, 13.44%), and the only one found in the three ecotopes studied. According to Brazil (2013), it is widely distributed in different ecological niches, especially in urban and rural areas. Because it is involved in the transmission of a disease of great social and economic relevance, and also because it is added to factors such as poor basic sanitation and accumulation of organic matter, it constitutes determining aspects for the maintenance of the vector in the environment. This data is very relevant, as *L. longipalpis* is considered the main vector of *L. infantum*, and although detected in smaller numbers in our study, it was found in all sampled points, being an important risk factor for the emergence of new cases in animals and humans. Therefore, entomological monitoring of potential *L. infantum* vectors is an important tool to prevent the spread of the disease.

In Santa Maria, VL cases have been reported since 1985, and two HVL cases were reported in 2021. However, until entomological studies were carried out, no sand fly species had been described in the region. In our study, all captured species were recorded in the state of RS; among these, *M. migonei* and *P. fischeri* are epidemiologically important because they have been implicated in *L. braziliensis* transmission, the main etiological agent of CL in Brazil (Shimabukuro & Galati, 2011). In several additional studies, they were implicated in infection with the etiological agent of VL, *L. infantum* (Guimarães et al., 2016).

In Santa Maria, *P. fischeri* (76/119, 63.86%) was the most frequently found species; however, it was only captured in the P3 ecotope, which is adjacent to a native forest environment. According to Rangel & Lainson (2003), *P. fischeri* has wild habitats, and its highest occurrence has been observed in areas of recent deforestation, especially where human habitation occurs. The species was naturally infected with *Leishmania* (*Viannia*) (Young & Duran, 1994) and *L. braziliensis* (Lana et al., 2015). Rêgo et al. (2020) reported the first molecular detection of *L. infantum* on *P. fischeri* in Porto Alegre and Galvis-Ovallos et al. (2021) confirmed its natural infection with *L. infantum* promastigotes in Embú das Artes, SP, focusing on CanL and HVL; this suggests it is a vector of this etiological agent, but does not confirm the vector potential (Alcover et al., 2012).

*Migonemyia migonei* was the second most common species (23/119, 19.32%) captured in the ecotope B (predominantly urbanized environment) and C (adjacent to native forest). This proves the ambience of this species, which was previously predominantly wild in urban environments. Guimarães et al. (2016) stated that this species is highly susceptible to *L. infantum* development, making it a permissive vector. According to Rangel & Lainson (2003), *P. fischeri* and *M. migonei* are notably anthropophilic, and according to Aguiar & Medeiros (2003), can also be captured in residual forests in marginal areas of cities, in annexes for domestic animals, and internal walls of human homes.

The main VL-transmitting species in Brazil, *L. longipalpis*, was the third most abundant in this study (16/119, 13.44%), and the only one found in all three surveyed ecotopes. According to Brazil (2013), it is widely distributed in several ecological niches, especially in urban and rural areas, where it has successfully established and proliferated, mainly due to anthropological environmental changes. This data is very relevant, as *L. longipalpis* is considered the main vector of *L. infantum*, and although detected in smaller numbers in our study, this vector was found at all sampled points. Therefore, entomological monitoring of potential vectors of *L. infantum* is crucial.

According to Aguiar & Medeiros (2003), *Brumptomyia* species are not important in leishmaniasis epidemiology. These sandflies are usually found in wild environments with leaves lying on the ground and reported in Dasipodidae (armadillo) burrows due to their food preferences. In our study, only two specimens of this genus were captured in the C ecotope.

*Psathyromyia lanei* (2/119, 1.68%) has wild and semi-domestic characteristics, inhabiting hollows and treetops in marginal areas and annexes of domestic animals. It is not associated with leishmaniasis transmission (Aguiar & Medeiros, 2003). This species was found only in C, which has all of these favorable conditions for its survival. Regarding insect seasonality, climatic factors, such as temperature, humidity, and rainfall, have a variable influence on sand fly populations, depending on the area studied (Dias et al., 2007). According to Souza et al. (2017), the temperature tends to be lower at the beginning of winter, causing a drop in the sand fly populations. However, our study also did not detect sanflies at higher temperatures, close to 25 °C (january 2021 and 2022). One potential explanation is “La Niña” phenomenon, which occurred both in 2021 and 2022 in RS, triggering long periods of drought, with rainfall below the historical average for the period. This may have negatively influenced the vector density, where the immature stages of these insects need organic matter and moisture to develop (Franke et al., 2002). Possibly, the intensity of rainfall as well as temperature variation may have influenced the entomological collection of vectors.

PCR analysis indicated that the natural infection rate in sand flies may be low due to many factors, for example, resisting digestion by interfering with digestive enzymes (Telleria et al., 2010). In addition, *Leishmania* secrete a myoinhibitory peptide that interrupts hindgut peristalsis, delays fecal elimination, and increases the persistence of the parasite within the insect (Vaidyanathan, 2004). *Leishmania* cause damage to the stomodeal valve, interfering with the blood ingestion process, which can often lead to vector death (Volf et al., 2004). Although the PCR results of this study were negative, it is important to highlight that the study of these vectors is essential for understanding disease epidemiology in the region.

This study reinforces the evidence of autochthonous transmission of both VL and HVL and includes the municipality as a possible transmission area for CL, although such cases have not yet been reported. Based on this, we recommend carrying out entomological surveys covering more areas of the city and reinforce that preventive measures be adopted by public authorities, such as vector control, and by dog owners, such as using repellent collars.

This study verified that the sand fly fauna in the municipality of Santa Maria, central RS, is diverse, with the *P. fischeri*, *M. migonei*, *L. longipalpis*, *Bumptomyia* sp., and *P. lanei* being present. We found specimens of epidemiological interest that have not been previously described in the region, with a predominance of *P. fischeri*, *M. migonei,* and *L. longipalpis*. Based on these results and the detection of three sand fly species associated with VL and CL transmission, Santa Maria, RS represents an important focus for these diseases, both in dogs and humans. It is therefore important that sanitary measures are adopted, with the aim of providing information to the inhabitants of the region, as well as the implementation of public policies aimed at reducing insect proliferation, such as vector control and the implementation of basic sanitation. From this, other studies can be carried out in the municipality to identify the parasite and compare it with infections in humans and animals. In addition, measures may include health education in the affected neighborhoods, provision of repellent collars, as well as responsible ownership and custody of dogs and implementation of policies to prevent, control and combat the disease through the Sistema Único de Saúde.
